# Evolutionary online behaviour learning and adaptation in real robots

**DOI:** 10.1098/rsos.160938

**Published:** 2017-07-26

**Authors:** Fernando Silva, Luís Correia, Anders Lyhne Christensen

**Affiliations:** 1 Bio-inspired Computation and Intelligent Machines Lab, 1649-026 Lisboa, Portugal; 2 BioISI, Faculdade de Ciências, Universidade de Lisboa, 1749-016 Lisboa, Portugal; 3 Instituto de Telecomunicações, 1049-001 Lisboa, Portugal; 4 Instituto Universitário de Lisboa (ISCTE-IUL), 1649-026 Lisboa, Portugal

**Keywords:** online evolution, learning, fault tolerance, real robots

## Abstract

Online evolution of behavioural control on real robots is an open-ended approach to autonomous learning and adaptation: robots have the potential to automatically learn new tasks and to adapt to changes in environmental conditions, or to failures in sensors and/or actuators. However, studies have so far almost exclusively been carried out in simulation because evolution in real hardware has required several days or weeks to produce capable robots. In this article, we successfully evolve neural network-based controllers in real robotic hardware to solve two single-robot tasks and one collective robotics task. Controllers are evolved either from random solutions or from solutions pre-evolved in simulation. In all cases, capable solutions are found in a timely manner (1 h or less). Results show that more accurate simulations may lead to higher-performing controllers, and that completing the optimization process in real robots is meaningful, even if solutions found in simulation differ from solutions in reality. We furthermore demonstrate for the first time the adaptive capabilities of online evolution in real robotic hardware, including robots able to overcome faults injected in the motors of multiple units simultaneously, and to modify their behaviour in response to changes in the task requirements. We conclude by assessing the contribution of each algorithmic component on the performance of the underlying evolutionary algorithm.

## 1. Introduction

Robots have significant potential to replace manned machines and to carry out tasks in environments that are either remote or hazardous, such as space, underground or deep sea. However, to create intelligent, reliable, mobile robots that can effectively operate in a wide variety of environments, the issue of *limited learning ability* of robots must be addressed [1]. The control system of mobile robots is typically based on predefined rules that remain fixed during task execution, regardless of what robots encounter in the environment. As a result, mobile robots tend to be brittle: they are unable to adapt to unforeseen changes in environmental conditions (e.g. changes in terrain or unexpected obstacles) or internal conditions (e.g. drift or permanent failure in sensors and/or actuators), which limits their versatility and usefulness. The capacity to learn and adapt during task execution is therefore crucial for mobile robots.

One radical and open-ended approach to learning and adaptation in robotic systems is *online evolution*. Online evolution employs evolutionary computation, a nature-inspired approach that mimics Darwinian evolution. Instead of a robot engineer manually programming the robots to carry out a mission, an evolutionary algorithm is executed on board each robot in order to create and continuously optimize its behavioural control logic. The evolutionary algorithm is executed autonomously without any external supervision or human interaction. Online evolution thus has the potential to automatically generate the artificial intelligence that controls each robot, which can give robots the capacity to cope with unforeseen changes.

Despite the potential for automatic robot learning, online evolution is not frequently employed in real-robot scenarios for a number of reasons. First, online evolution typically requires several hours or days to synthesize solutions to any but the simplest of tasks [2]. Second, one common assumption in the field is that online evolution enables continuous learning and adaptation to previously unforeseen circumstances. However, successful demonstrations that this assumption holds true are scarce. In simulation, only a small number of ad hoc experiments have been carried out [3–6]. On real robots, successful demonstrations of the adaptive capabilities of online evolution have been limited to changing the position of a light source in a dynamic phototaxis task [3].

In this article, we focus on two key aspects: (i) if and how online evolution can enable autonomous learning and adaptation in real single robots and multi-robot systems and (ii) how to enable effective online evolution of behavioural control, including coordinated behaviours when groups of robots are considered, that is, whether or not robots can learn to solve different classes of tasks within a time frame of 1 h or less. We carry out online evolution on real Thymio II robots, and we use the odNEAT evolutionary algorithm [4], which simultaneously optimizes the weights and the topological structure of artificial neural network-based controllers.

Our first research question refers to how to best seed online evolution so that controllers can be efficiently evolved. We assess how online evolution from an initial random population in real robotic hardware compares with online evolution seeded with controllers pre-evolved in simulations with varying degree of fidelity. We then assess the relations between simulation fidelity, task complexity and the effort required to readapt online controllers that do not transfer well from simulation to reality. Although online readaptation of controllers was hinted at in the 1990s [7], it was only studied in an ad hoc manner. Particularly, online readaptation was investigated without regard to how accurate a simulation needs to be in order to provide an effective bias, and in a single task (navigation and obstacle avoidance by a single robot). Our second research question is related to the unexplored adaptation and learning potential of online evolution. We therefore set up a series of adaptation experiments, in which we change the task requirements during the experiment, and we inject simulated faults in the motors of multiple robots simultaneously.

We study two single-robot tasks: (i) integrated navigation and obstacle avoidance, and (ii) homing towards a target area; and one collective robotics task, namely aggregation. The integrated navigation and obstacle avoidance task is used to analyse the performance of online evolution when two conflicting objectives have to be learned. The task implies an integrated set of actions, and thus a trade-off between avoiding obstacles and maintaining speed and forward movement. Navigation and obstacle avoidance is therefore essential for autonomous robots operating in real-world environments, and it provides the basis for more complex behaviours such as path planning [8].

The second task, homing, is an extension of navigation tasks in which a single robot starts from a random position, and must search for and navigate to a small target area located in the centre of the environment. The third task, aggregation, is a multi-robot task in which dispersed robots must move close to one another so that they form and remain in a single group. Aggregation thus combines different aspects of collective robotics tasks, namely distributed search, coordinated movement and cooperation. Aggregation is also related to a number of real-world robotics tasks. For instance, self-assembly and collective transport of heavy objects require the robots to aggregate at the site of interest [9]. In our aggregation task, we divide the arena into three equally sized areas. A group of three robots must then aggregate in one of the two areas at the extremes of the arena, similarly to collective decision-making scenarios used to investigate the best-of-*n* decision problem in robot collectives [10].

We show that our approach to online evolution enables efficient synthesis of controllers from random solutions in real robotic hardware, and readaptation of controllers after they are transferred from simulation to reality. We also demonstrate for the first time the adaptive capabilities of online evolution in real robotic hardware, as robots effectively learn new behaviours and adapt to faults in the motors so as to carry out the required tasks. To conclude, we analyse how our results are influenced by the choice of the underlying online evolution algorithm, and we show that the performance of odNEAT is supported by evolutionary mechanisms seldom adopted in online evolution algorithms, such as explicit speciation of solutions with differing complexity, and recombination of controllers with different structure [4]. In summary, our research is a contribution towards scaling online evolution to real-world applications, in which robots have to take on tasks in potentially dynamic environments, and to the development of more effective online evolution algorithms.

## 2. Material and methods

Our experimental methodology is defined by three key components, namely: (i) specification of the task setup, (ii) evolution in simulation, and (iii) evolution in real hardware. Each component is described below. Experiments were carried out in a square arena. The size of the simulated arena for evolution of the seed controllers and of the real arena was chosen to be 100×100 cm. The real arena was surrounded by wooden walls. Each simulated robot and each real robot was controlled by a discrete time recurrent neural network [11]. The inputs of the neural network were normalized sensor readings, and the outputs of the network controlled the robot’s actuators. Every 100 ms, robots executed a control cycle, in which they updated sensor readings and actuation values.

*Specification of the task setup*:

(i) Construction of the environment for the real-robot experiments, and modelling of an environment with similar characteristics in simulation.
(ii) Configuration of the robots’ sensors and actuators, particularly the processing of raw sensory data to extract information that can be fed to the robots’ neural controllers.
(iii) Modelling of robot sensors and actuators in simulation. As sensors are often the main source of stochasticity in robots [7], we used three complementary methods to model sensory inputs: taking samples from the real robots’ sensors [12], introducing a conservative form of noise in simulated sensors [12] and perfect readings, that is, without any stochasticity associated. For the simulated actuators, we built a differential-drive kinematics model, adjusted the model based on observations of the motor speeds of real robots and applied a conservative amount of noise. Noise in simulated sensors and actuators corresponded to a random Gaussian component within 5% of the sensor saturation value or of the current actuation value. It should be noted that our approach to modelling robot sensors and actuators in simulation does not preclude the use of more elaborate methodologies, such as the *transferability approach* [13]. The transferability approach relies on multiobjective optimization in which controllers are evaluated based on their performance in simulation and on the real robots; it uses a surrogate model that is updated periodically by evaluating candidate solutions in real hardware. The goal is to learn the discrepancies between simulation and reality, and to constrain evolution in order to avoid behaviours that may not transfer effectively from simulation to reality.
(iv) Definition of the fitness function. In our experiments, we resorted to internal fitness functions [14], which are based on information that is available to the robot (e.g. through its sensors or state of the motors). In this way, fitness assessment is self-contained, as an individual robot does not require any external devices to determine its performance.

*Evolution in simulation*: Our simulation-based experiments were conducted using JBotEvolver [15], an open-source, multi-robot simulation platform and evolutionary framework. Each robot was controlled by an artificial neural network. To optimize controllers in simulation, we used the NEAT algorithm [16], a widely adopted generational evolutionary algorithm that simultaneously optimizes the connection weights and the topological structure of neural networks. A full description of the algorithm is given in [16]. We conducted experiments in nine evolutionary setups (three complementary methods to model sensory inputs for each of the three tasks). Each evolutionary setup consisted of 30 independent evolutionary runs conducted in simulation. In each simulation-based run, the evolutionary process optimized a population of 100 controllers for 100 generations. At each generation, a controller’s performance was given by the mean fitness of 10 simulations with varying conditions (e.g. randomized initial position and orientation, amount of noise injected at every simulation step in sensors and/or actuators). After each evolutionary run ended, we carried out a post-evolution evaluation. The top controller of each generation was evaluated in 100 simulations (total of 3000 controllers evaluated without evolution) to obtain a more precise estimate of the performance. Based on the post-evolution evaluation results, we identified the highest performing controller of each evolutionary setup.

**Figure 1.**
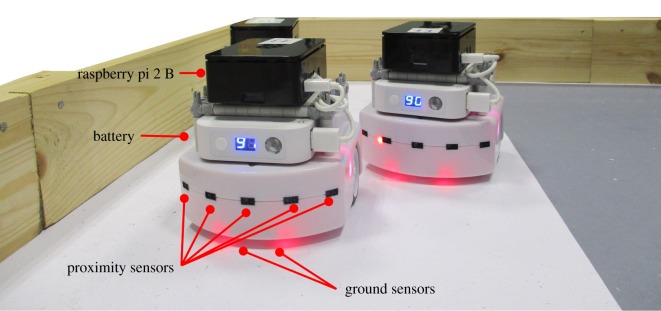
Real robotic platform. Each Thymio II robot is extended with a Raspberry pi 2 B single-board computer. The Thymio II robot has seven infrared proximity sensors (five in the front, two in the back) and two infrared ground sensors, among others.

*Evolution in real hardware*: To conduct our real-robot experiments, we used Thymio II robots (figure 1). Each robot was extended with a Raspberry Pi 2 B single-board computer. In multi-robot experiments, the robots formed an IEEE 802.11g ad hoc wireless network, and communicated with one another by broadcasting UDP datagrams. For controller evolution, we used the odNEAT algorithm [4], which we describe in the following section.

In the experiments presented in this article, online evolution was either conducted from random solutions on real robotic hardware, or seeded with controllers previously evolved in simulation. For each real-robot experimental setup, five independent runs were conducted. Each run lasted 1 h. Individual controller evaluations on real robots lasted 30 s in the navigation and obstacle avoidance task, and 25 s in the homing and aggregation tasks (individual evaluations in these two tasks were preceded by a 5 s long random walk), allowing for a total of 120 evaluations per experiment.

### 2.1. Online evolution of control with odNEAT

odNEAT is distributed across multiple robots that exchange candidate solutions to the task. Specifically, the evolutionary process is implemented according to a physically distributed island model. Each robot optimizes an internal population of genomes (directly encoded artificial neural networks) through intra-island variation, and genetic information between two or more robots is exchanged through inter-island migration. The genomes are probabilistically exchanged between robots at every control cycle. The exchange procedure promotes the propagation of genomes representing networks with novel forms of topological structure and competitive performance levels (see [4] for details). In this way, each robot is potentially self-sufficient and the evolutionary process opportunistically capitalizes on the exchange of information between multiple robots for collective problem solving [4].

odNEAT starts with fully connected artificial neural networks with no hidden neurons, that is, with each input neuron connected to every output neuron. Throughout evolution, topologies are gradually complexified by adding new neurons and new connections through mutation. In addition, the internal population of each robot implements a niching scheme comprising speciation and fitness sharing, which allows the robot to maintain a healthy diversity of candidate solutions with distinct topologies [4]. New controllers are synthesized via selection of a parent species and two genomes from that species (the parents), crossover of the parents’ genomes and mutation of the offspring. Mutation is both structural and parametric, as it adds new neurons and new connections, and optimizes parameters such as connection weights and neuron bias values.

### 2.2. Navigation and obstacle avoidance

In the navigation and obstacle avoidance task, a robot has to simultaneously move as straight as possible, maximize wheel speed and avoid obstacles. In a confined environment, such as our enclosed arena, this task implies an integrated set of actions, and consequently a trade-off between avoiding obstacles and maintaining speed and forward movement.

The input layer of a neural network-based controller for the navigation and obstacle avoidance task has seven neurons. The input neurons receive the readings from the seven horizontal infrared sensors for obstacle detection (five in the front, two in the back), normalized to the interval [0, 1]. The output layer is composed of two neurons. The values of the output neurons are linearly scaled from [0, 1] to [−1, 1] to set the signed speed of each wheel. The fitness score *f*_*nav*_ of a controller is given by Floreano & Mondada [17]:

2.1 $$f_{\mathrm{nav}}=\sum_{i=1}^{T}\frac{V\cdot \left(1-\sqrt{\Delta v}\right)\cdot (1-d_{o})}{T},$$

where *T* is the length of an experiment in control cycles, *V* ∈[0,1] is the normalized absolute sum of rotation speeds of the two wheels, *Δv*∈[0,1] is the normalized absolute value of the algebraic difference between the signed speed values of the wheels and *d*_*o*_ is the highest activation value of the infrared sensors for obstacle detection. We see that *d*_*o*_ takes values from 0 (no obstacle in sight) to 1 (collision with an obstacle). The three components encourage, respectively, motion, straight line displacement and obstacle avoidance.

### 2.3. Homing

**Figure 2.**
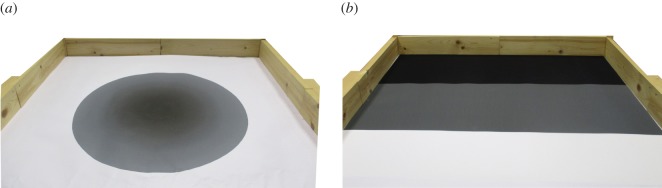
Real environment for the homing experiments (*a*), and for the aggregation and adaptation experiments (*b*). The environment for the aggregation and adaptation experiments is composed of three areas, namely a black area (top), a grey area (middle) and a white area (bottom).

*Homing* is an extension of navigation tasks in which a robot must move towards a point of interest in the environment. In our homing task, a single robot has to actively search for and navigate to a small target area located in the centre of the environment. The target area has a radius of 5 cm. To allow Thymio robots to sense the target area, we sampled the ground sensors’ response to different colour components, and created a circular grey gradient surrounding the target area (figure 2*a*). This configuration allows a robot to sense the target area at a distance of up to 25 cm.

The input layer of a controller for the homing task has eight neurons. The input layer receives the normalized readings of the seven horizontal infrared sensors and the robot’s proximity to the target area, scaled to a value between 0 (target area not in range) and 1 (robot is in the target area). The proximity value is computed based on the mean value of the readings from the two infrared ground sensors. Similarly to the navigation and obstacle avoidance task, the output layer contains two neurons for setting the signed speed of each wheel.

The position of the robot is randomized at the beginning of every evaluation by executing a 5 s long random walk. Controllers are then scored based on the robot’s proximity to the exterior part of the target area, up to 25 cm, for the remaining 25 s of the evaluation period:

2.2 $$f_{\mathrm{homing}}=\sum_{i=1}^{T}\frac{p_{t}}{T},$$

where *T* is the maximum number of control cycles and *p*_*t*_ is the robot’s proximity to the target area (linear inverse of the distance).

### 2.4. Collective decision-making: aggregation in a non-determined area

In an aggregation task, dispersed robots must move close to one another so that they form a single group. In the aggregation task used in our study, we divide the arena into three equally sized areas (figure 2*b*). A group of three robots must aggregate in one of the two areas at the extremes of the arena, denoted as area A and area B.

The input layer of each controller for the aggregation task is composed of 11 neurons: seven neurons receive the normalized readings of the horizontal infrared sensors for obstacle detection; two neurons receive binary inputs indicating whether the robot is in area A or not, and whether in area B or not; and two neurons receive the ratio of other robots in area A and in area B. The binary inputs are computed based on the mean value of the readings from the two infrared ground sensors. Each robot additionally broadcasts these inputs to other robots, which allows receiving robots to compute the ratio of robots in area A and in area B. As in the previous two tasks, the output layer is composed of two neurons that control the speed of the wheels.

Similarly to the homing task, the position of robots is randomized at the beginning of each controller evaluation. A controller is scored according to the area in which it is located and the areas in which other robots are located:

2.3 $$f_{\mathrm{agg}}=\sum_{i=1}^{T}\frac{f_{\mathrm{option}}}{T},\;f_{\mathrm{option}}=\left\{ \begin{array}{ll} 1+\Delta_{\mathrm{area}} & \text{if robot is in area A or area B},\\ 0 & \mathrm{otherwise}, \end{array}\right.$$

where *T* is the duration of the experiment in number of control cycles and *Δ*_*area*_ is the number of other robots in the same area.

### 2.5. Adaptation experiments

*Task adaptation experiments*: In these experiments, we first evolve controllers to solve the aggregation task, in which robots must aggregate either in area A or in area B, and we then change the task so that robots have to aggregate in the centre of the arena (figure 2*b*). A controller is scored based on

2.4 $$f_{\mathrm{agg}\_\mathrm{centre}}=\sum_{i=1}^{T}\frac{f_{\mathrm{centre}}}{T},\;f_{\mathrm{centre}}=\left\{ \begin{array}{ll} 1+\Delta_{\mathrm{centre}} & \text{if robot is in centre area},\\ 0 & \mathrm{otherwise}, \end{array}\right.$$

where *T* is the duration of the experiment in number of control cycles and *Δ*_*centre*_ is the number of other robots in the centre area. While intuitively simple, this change in the task requirements forces robots to radically modify their response to key sensory inputs.

*Fault injection experiments*: The ability to tolerate and/or actively overcome faults is essential for improving the reliability of robot systems [18]. In the fault injection experiments, we simulate faults in the motors of real robots evolved to solve the aggregation task. We conduct three sets of experiments, in which we inject faults in the motors of *N*={1,2,3} robots. The fault injection procedure consists of: (i) selecting the required number of robots, (ii) randomly choosing either the left or the right wheel of each of the *N* robots and (iii) randomly limiting the wheel speed to 50–75% of its maximum value. Such faults are analogous to failures in servo drives and motor-amps of mobile robots operating for long periods of time [19].

In both the task adaptation and the fault injection experiments, we resume the evolutionary process after the respective changes take place. Specifically, robots first evolve for one hour to solve the initial version of the aggregation task, as described in the previous section. We then select the group of real robots with highest fitness on average, and allow the robots to evolve for another hour in order to adapt to changes.

### 2.6. Performance analysis and treatments

We compare results along three dimensions: (i) fitness, that is, the performance or quality of a controller, (ii) percentage of successful controller evaluations per experiment, and (iii) behaviour distance, that is, how distinct the actions performed during task execution by one or more reference controllers are with respect to the actions performed by other controllers. As robots are repositioned from one evaluation to the next, the percentage of successful evaluations is a more consistent metric than the traditional number of evaluations until the first successful controller evaluation, thus providing more accurate indications of the ability of robots to learn and adapt.

We have empirically determined performance-based criteria to distinguish between successful controller evaluations and failed controller evaluations. In the integrated navigation and obstacle avoidance task, a controller evaluation is considered to be successful if the fitness score *f*_*nav*_ of the controller is above 0.5 (e.g. the robot effectively avoids obstacles and navigates at least at half of the maximum speed, or moves at maximum speed and can avoid obstacles before they are at a distance of half of the sensor range). In the homing task, a controller evaluation is considered successful if the robot is able to find the target area, and stay in it for at least three consecutive seconds. Similarly, an evaluation in the aggregation task is considered successful if all robots in the group can coordinate and remain in the same area for at least three consecutive seconds. In the homing task and in the aggregation task, the controller success versus failure analysis is, respectively, complemented by the amount of time that a robot has spent in the target area, and by the amount of time that a group of robots has spent aggregated.

To compare behaviours and thus compute behaviour distances, a characterization of individual controllers according to behaviour is needed [20,2]. We resorted to a domain-independent behaviour characterization based on the mapping between sensors and actuators. Specifically, the behaviour metric is based on the set of sensor readings and actuation values *ϑ* normalized into [0,1]. The binary version *ϑ*_*bin*_ of *ϑ* is then computed such that each *ϑ*_*bin*_(*i*)∈*ϑ*_*bin*_ is defined as 1 if *ϑ*(*i*)> 0.5 and 0 otherwise. The distance between two behaviours is computed as the Hamming distance between them. This metric has shown to be at least as efficient as domain-dependent behaviour metrics [21]. A complete description of the method is given in [21]. To analyse how the evolutionary process proceeds, we construct two-dimensional *behaviour–fitness maps*, which relate behaviour distance with task performance. It should be noted that our concept of behaviour-fitness map differs from the concept of *behaviour performance map* presented in [22], which is used to record the highest-performing individual found during evolution for each point in a discretized version of the behaviour space.

We use the two-tailed Mann–Whitney test to compute significance of differences between sets of results because it is a non-parametric test, and therefore no strong assumptions need to be made about the underlying distributions. When multiple comparisons are performed, we adjust the *ρ*-value using the two-stage Hommel method [23]. Success rates are compared using the two-tailed Fisher’s exact test [24].

## 3. Results

The presentation of experimental results is divided into three subsections. In the first subsection, we report on how to best seed online evolution in real hardware. In the second subsection, we describe if and how online evolution can enable adaptation to changes in the task requirements and to faults injected in the motors of multiple robots. In the third subsection, we detail and discuss how the properties and different algorithm components of odNEAT influence controller synthesis.

### 3.1. Evolution of control in real robotic systems

In this section, we evaluate how online evolution from an initial random population in real hardware, henceforth called *baseline* evolution, compares with online evolution seeded with controllers pre-evolved in simulation. We address three key research questions: (i) to what extent the online evolutionary process can be accelerated by being seeded with controllers pre-evolved in simulation, (ii) how accurate a simulation needs to be in order to provide an effective bias, and (iii) how much evolutionary effort is required to adapt pre-evolved controllers that do not transfer well from simulation to real robotic hardware.

We evolved seed controllers in simulations with varying degree of fidelity by using three complementary methods to model sensory inputs: relying on samples taken from the real robots’ sensors [12]; introducing a conservative form of noise in simulated sensors [12]; and perfect readings, that is, without any noise or stochasticity associated. The highest performing controller of each simulation-based setup (real samples, conservative noise, no noise) is subsequently used in a set of real-robot experiments, in which it is specified as the initial controller in the online evolution algorithm [4] (see electronic supplementary material, Text S1, for experiments on how the highest performing controllers found in simulation transfer to real robotic systems).

#### 3.1.1. Influence of the seeding process on controller evolution and readaptation

In the navigation and obstacle avoidance task, the mean percentage of successful controller evaluations per robot is 38.0±16.1% for the baseline setup, 9.5±9.0% for the no-noise setup, 6.5±12.3% for the conservative noise setup and 85.8±3.6% for the real samples setup. The percentage of successful controllers evolved with the real samples method is significantly superior to the percentage of successful controllers evolved with other methods (*ρ*<0.0001); the baseline method also outperforms the no-noise method and the conservative noise method (*ρ*<0.0001).

**Figure 3.**
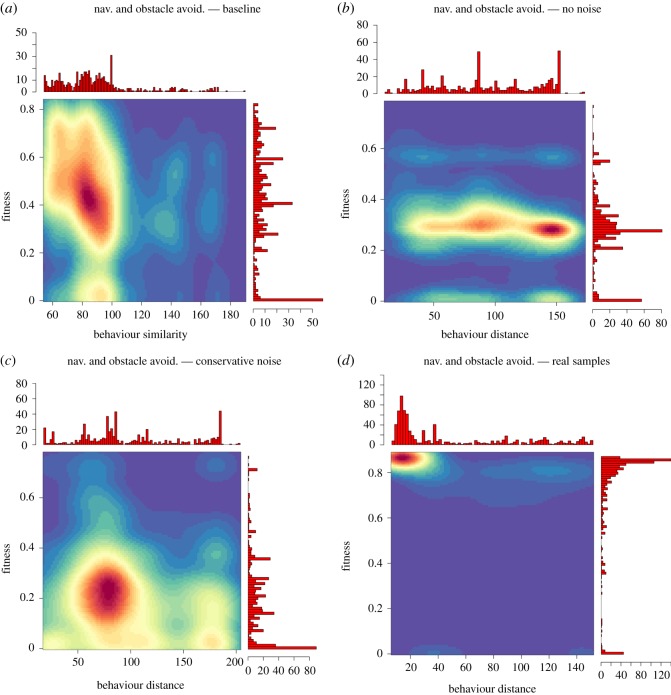
Behaviour–fitness map for the navigation and obstacle avoidance tasks. Behaviour distance is relative to the initial controllers: a randomly generated controller for the baseline setup (*a*), and the seed controller for the no-noise setup (*b*), conservative noise setup (*c*) and real samples setup (*d*). The fitness score of a controller is a linear combination of two components, specifically fast and forward movement, and avoiding obstacles in sensor range. The two components are normalized in the interval [0,1] and measured over all control cycles of the robot (for a given evaluation). We empirically determined that fitness scores higher than 0.9 cannot be obtained in our enclosed arena.

Figure 3 shows two-dimensional behaviour–fitness maps, which relate behaviour distance with task performance, for the four setups of the navigation and obstacle avoidance task (baseline, controllers pre-evolved in simulations using perfect sensor readings, a conservative form of noise in simulated sensors, and samples from real robots). To elucidate on how evolution proceeds in the behaviour space, *behaviour distance* is relative to the initial controllers. The maps show that the progress of the online evolutionary process through the behaviour space is strongly influenced by the choice of seeding method. In the baseline setup, the evolutionary process mainly discovers a certain class of behaviours (those with behaviour distance between 80 and 100; figure 3*a*,*c*). In the no-noise setup, evolution synthesizes behaviours at varying distances from the seed controllers, but can rarely reach high-fitness regions. This result indicates that seeding evolution with controllers from the no-noise setup biases the search towards uninteresting regions of the behaviour space. In these regions, high-performing controllers are challenging to synthesize, hence the comparatively low rate of successful controller evaluations.

If evolution is seeded with controllers from the conservative noise setup, it performs similarly to when it is seeded with controllers from the no-noise setup. However, the evolutionary process performs a more localized search for solutions in behaviour space (behaviour distances approx. between 50 and 100). This result indicates that the bias induced by seed controllers is stronger, which in turn increases the readaptation effort required to evolve effective controllers. The final map shows that if evolution is seeded with controllers from the real samples setup, it is able to consistently synthesize high-performing solutions to the task (figure 3*a*).

The results presented in this section indicate that simulations with different sensor models impose distinct constraints on the online evolutionary and readaptation process. In the following sections, we assess if and how the results generalize to more challenging tasks (e.g. that require searching for a target area or group coordination), specifically the homing task and the aggregation task.

#### 3.1.2. Challenging the reality gap with online evolution

In the homing task, the mean percentage of successful evaluations per robot is 3.7±0.7% for the baseline setup, 6.3±2.0% for the no-noise setup, 3.8±2.9% for the conservative noise setup and 4.2±1.7% for real samples setup. Differences are not statistically significant across any comparison (*ρ*>0.05). While the mean percentages of successful evaluations per robot are comparatively lower in the homing task than in the integrated navigation and obstacle avoidance task, the online evolutionary process is still able to consistently synthesize a considerable set of effective controllers in every run: approximately four to eight solutions to the task per 1 h experiment.

**Figure 4.**
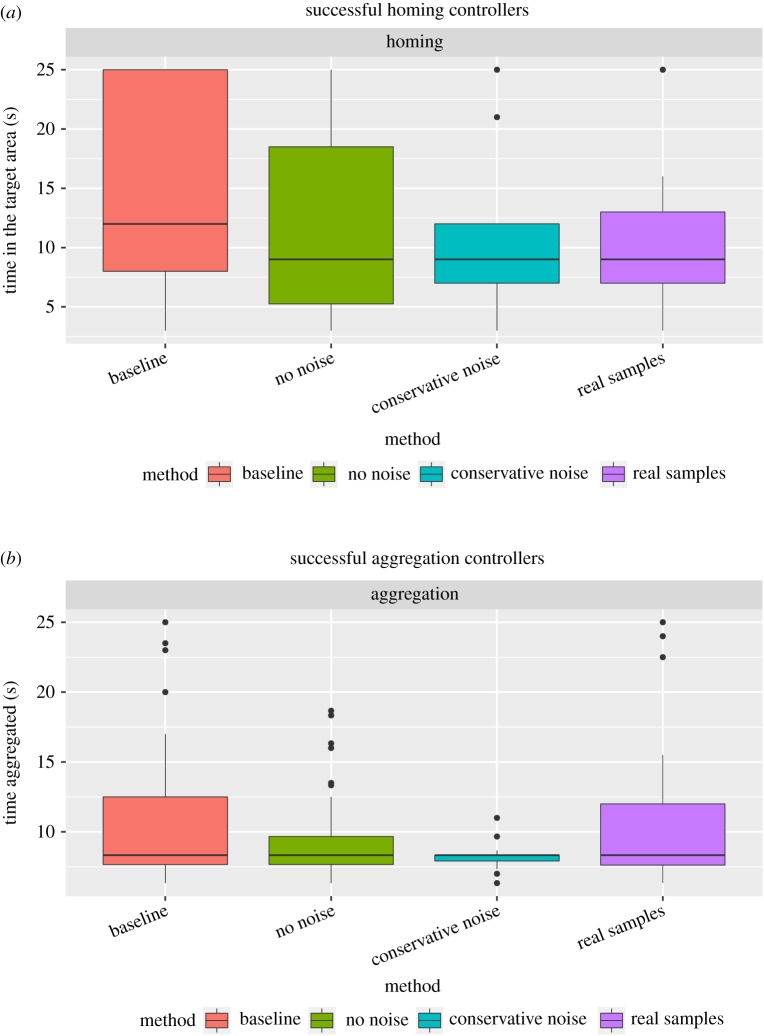
Successful controllers for the homing and aggregation tasks. (*a*) Time spent in the target area by the controllers that can successfully carry out the homing task. (*b*) Amount of time that successful aggregation controllers spent in the same area.

Figure 4*a* shows the amount of time spent in the target area by the controllers that can successfully carry out the homing task. The median amount of time in the target area is 12 of 25 s in the baseline set-up, and 9 of 25 s for other setups. These combined results furthermore show that: (i) effective solutions to the task can be evolved from random solutions on real robots and (ii) online evolution is able to readapt controllers that do not transfer well from simulation to real robotic hardware. Importantly, the fact such readaptation actually occurs in one hour or less, which is the time frame considered in our experiments, challenges the view that completing the optimization process in a real robot is only meaningful if solutions found in simulation are close to solutions in reality, that is, if the reality gap is sufficiently narrow.

#### 3.1.3. Evolution of collective behaviours

In the aggregation task, the mean percentage of successful evaluations per group of robots is 11.5±4.0% for the baseline setup, 14.8±11.2% for the no-noise setup, 8.7±5.3% for the conservative noise setup and 9.0±3.2% for the real samples setup. Differences are not statistically significant across all comparisons (*ρ*>0.05). Every 1 h experiment therefore consists of 10–18 groups of controllers able to simultaneously coordinate the behaviour of the respective robots in order to reach collective decision-making, namely aggregation in a non-determined area.

Figure 4(*b*) shows the amount of time that groups of three robots able to carry out the aggregation task spent in the same area. The median amount of time aggregated is approximately 8 of 25 s for all experimental setups. The highest performing groups of controllers, that is, those that aggregate the longest, are typically synthesized either in the baseline setup or in the real samples setup. These results demonstrate that online evolution allows robots to effectively evolve solutions to the aggregation task. To the best of our knowledge, this experiment is the first instance of online evolution of collectively coordinated behaviours with real robots. Previous studies involving multiple robots have been limited to individual tasks such as phototaxis [25], dynamic phototaxis [3], foraging [26], and a combination of foraging and phototaxis (robots can use a moving light source as an environmental aid) in which controllers are evolved in an on-board simulator [27].

### 3.2. Behaviour adaptation and fault tolerance in real robotic systems

In this section, we assess if and how robots can adapt and learn new behaviours when task requirements change and when faults are injected in the motors. The aggregation task is arguably the most challenging of the three tasks for evolution to find a solution for. Using the aggregation task, we devised two experimental setups to assess the ability of online evolution to adapt behavioural control, namely: (i) *task adaptation experiments*, in which we change the configuration of the aggregation task so that robots have to aggregate in the centre of the arena after learning to successfully aggregate in one of the extremes, which forces robots to respond to sensory information in a radically different way, and (ii) *fault injection experiments*, in which simulated faults analogous to failures in servo drives and motor-amps [19] are injected in the motors of *N*={1,2,3} of three robots after they have learned to solve the initial version of the aggregation task. The different configurations of the fault-injection experiments are called the *one-fault setup*, the *two-fault setup* and the *three-fault setup*.

#### 3.2.1. Task adaptation results

The mean percentage of successful evaluations per group of robots is 11.7±8.8% for the task adaptation experiments, which is comparable to that of the baseline setup, despite a higher standard deviation: 11.5±4.0% of successful evaluations. Differences in successful controller evaluations are not statistically significant (*ρ*>0.05). Importantly, the online evolutionary process maintains a comparable rate of successful *groups* of controllers before and after changes to the task take place, approximately 14 groups of controllers, which indicates effective readaptation.

**Figure 5.**
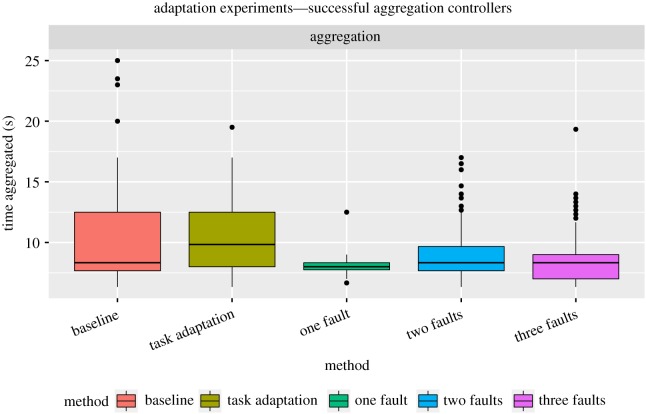
Successful controllers for the adaptation experiments. Amount of time that controllers spent in the same area.

The analysis of the amount of time that robots spent aggregated in the different adaptation experiments (figure 5) indicates that robots are able to effectively adapt to the new circumstances. In the task adaptation experiments, robots reach performance levels similar to those of baseline experiments: they learn to effectively aggregate in the centre area and thus to solve a new instance of the aggregation task. Importantly, the evolutionary process focuses mainly on optimizing a certain class of behaviours in order to readapt controllers to the new task (see electronic supplementary material, figure S3 for details).

#### 3.2.2. Fault injection results

The mean percentage of successful evaluations per group of robots is 3.0±1.3% for the one-fault setup (four groups of successful controllers), 11.3±9.9% for the two-fault setup (14 groups) and 14.7±7.9% for the three-fault setup (18 groups). Interestingly, the fault injection progressively leads to more successful evaluations as the number of faults increases. Successful controller evaluations are significantly higher in the baseline setup, two-fault setup and three-fault setup than in the one-fault setup (*ρ*<0.0001).

In the fault injection experiments, successful controllers cause robots to aggregate for a shorter amount of time when compared with the baseline setup (figure 5). The reason is that faulty robots, even after they learn to cope with a faulty wheel, are still limited in the speed they can achieve (from 50% to 75% of the maximum speed). As a result, robots require more time to find one another and aggregate. In the one-fault setup, only one robot is in a faulty state. The faulty robot has to learn to overcome the fault by itself, as controllers evolved by the other two robots in the group are optimized for non-faulty wheels, and cause the faulty robot to move in circles. In this way, the one-fault setup yields a comparatively lower percentage of successful controller evaluations per group of robots.

In the two-fault setup, the stochasticity of the fault injection causes the evolutionary process to commonly optimize behavioural control for a group of robots in which there is: (i) one non-faulty robot, (ii) one robot with a simulated fault in its left wheel and (iii) one robot with a simulated fault in its right wheel. Consequently, evolution focuses on behaviours that differ more from the initial controllers than those evolved in the one-fault setup (see electronic supplementary material, figure S3 for the behaviour–fitness map). The increased behaviour exploration is beneficial, as the performance levels of robots increase with respect to the one-fault setup both in terms of the successful controller evaluations, and of the amount of time that the group of robots was aggregated. The results of the three-fault setup are similar to those of the two-fault setup. Although the amount of time aggregated is comparable, both the percentage of successful controller evaluations and the behaviour distance to initial controllers are higher in the three-fault setup than in the two-fault setup.

### 3.3. Ablation studies

One key component in our experimental methodology is the underlying online evolution algorithm, odNEAT [4], which has been shown to outperform other state-of-the-art algorithms in simulation-based experiments [4]. odNEAT employs techniques seldom adopted in online evolution, including: (i) genotypic diversity mechanisms, particularly speciation and fitness sharing, which allow the algorithm to maintain a healthy diversity of genomes representing differing structures and network complexities throughout evolution, (ii) crossover between genomes representing networks with different topologies, which is supported by historical markers to enable meaningful recombination and (iii) a hybrid, island-model-like evolutionary structure: each robot optimizes an internal population of genomes through intra-island variation, and genetic information between two or more robots is exchanged through inter-island migration.

Using the aggregation task, we have ablated the main algorithm components of odNEAT. We conducted experiments in four distinct experimental setups: (i) without the genotypic diversity mechanisms (*no niching*), (ii) without the crossover operator (*no crossover*), (iii) with *no exchange* of genomes between robots and (iv) with a *minimal population* of size 1, which combines ablations (i), (ii) and (iii) because the internal population of each robot can only hold the genome corresponding to the controller that is executing. The minimal population ablation transforms odNEAT into a variation of the (1+1)-online algorithm [28], which was proposed after the classic (1+1) evolutionary strategy, and upon which multiple online evolution algorithms are based [3,4].

**Figure 6.**
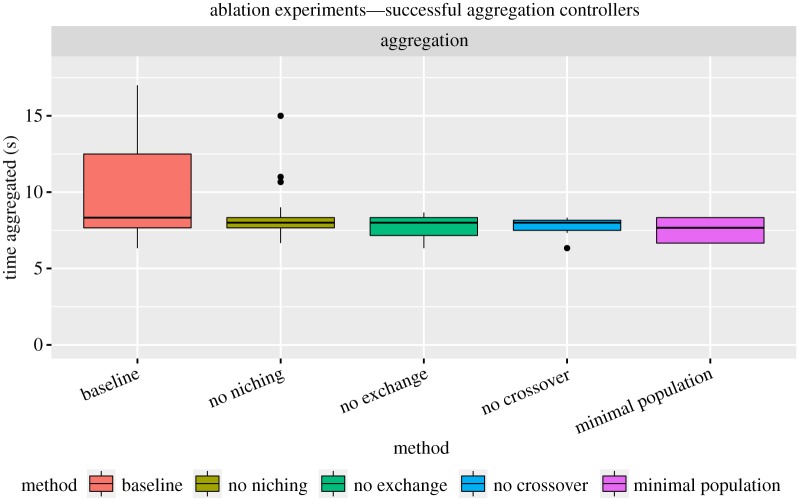
Successful controllers for the ablation experiments. Amount of time that robots have spent aggregated. The baseline setup, in which non-ablated odNEAT is used, yields five outliers above 18 s, namely 20 s, 23 s, 23.5 s, 23.5 s and 25 s (not shown for better reading of the plot).

The mean percentage of successful evaluations per group of robots is 11.5±4.0% for baseline evolution (non-ablated odNEAT, 14 groups of successful controllers), 5.7±1.7% when the niching scheme is ablated (seven groups), 3.2±1.8% when the crossover operator is ablated (four groups), 2.5±1.0% when the exchange of genomes is ablated (three groups) and 1.5±1.5% when the ablations are combined (minimal population ablation, two groups). The non-ablated version of odNEAT enables successful controller evaluations significantly more often than every ablated version (*ρ*< 0.05). Furthermore, the non-ablated version of odNEAT generates more effective controllers, which are able to form and remain in a single group for a larger amount of time (figure 6). The main conclusion is thus that each component of odNEAT has a significant contribution to the algorithm’s performance as an efficient online evolution algorithm.

## 4. Discussion

In our real-robot experiments, we successfully evolved behavioural control online to solve two single-robot tasks and one collective robotics task. In addition, we have demonstrated for the first time the adaptive benefits of online evolution in real robotic hardware: we have successfully shown that robots can adapt to changes in the task requirements and to faults in the motors. In this respect, follow-up studies of our contribution could conduct experiments similar to ours in laboratory conditions to investigate, for example, how pre-evolution in simulation influences not only learning to solve a given task (as in our first set of experiments), but also readaptation in the case of changes in environmental conditions and/or faults (as in our second set of experiments). However, in order to scale online evolution to real-world applications, the issues discussed below need to be addressed.

### 4.1. Increasing the performance of online evolution

In our experiments, we have considered a time frame of one hour for successful controller evolution in real robotic hardware, which allowed for a total of 120 controller evaluations per experiment. In the homing task, the mean percentage of successful controller evaluations is comparatively lower than in the other two tasks considered: from approximately 3.7% to 6.3% of successful controller evaluations, which corresponds to a set of four to eight successful solutions to the task per 1 h experiment. While the online evolutionary process is able to consistently synthesize a set of solutions to the homing task, there are different options to potentially increase the performance of evolution in terms of the percentage of successful controllers. In recent simulation-based contributions, we have developed two complementary approaches, namely: (i) a racing technique to cut short the evaluation of poor controllers based on the task performance of past controllers [29] and (ii) a novel paradigm called online hyper-evolution [30,31], which can both combine the benefits of different algorithms for controller generation over time, and *construct* algorithms by selecting which algorithmic components should be employed for controller generation (e.g. mutation, crossover, among others). Similarly to traditional evolutionary robotics approaches, online hyper-evolution can employ fitness-based search, behaviour-based search (novelty and/or diversity) and combinations of the two. Future work should assess how such techniques fare in real-robot online evolution of control.

### 4.2. Self-supervised behaviour learning

In the vast majority of online evolution algorithms, the evolutionary process takes place at regular intervals: each controller is assessed for a fixed, predefined amount of time, after which a new controller is generated. However, for online evolution to scale to real-world applications, more sophisticated techniques to *control* the evolutionary process and determine *when* robots should learn new behaviours are necessary. In real-world tasks, continuously producing new controllers can potentially harm individual task execution, because evaluation of inferior controllers amounts to poor task performance, and disrupt group behaviours (e.g. one robot changes from a high-performing controller to a low-performing controller when a robot collective is pushing an object towards a target area) [4].

A potential solution is enabling robots to autonomously enter new evolutionary and adaptation periods by *self-supervising* their behaviour learning process. One field of research from which inspiration can be sought is that of *artificial curiosity* [32], in which agents have an internal drive that pushes them towards situations in which, for example, reward-based learning progress is maximized. Originally introduced in the reinforcement learning domain, the artificial curiosity framework has been used for active learning tasks [33].

### 4.3. Scaling the evolutionary process to more complex tasks

Online evolution is well-aligned with a long-standing goal in artificial intelligence and robotics: synthesizing agents that can effectively learn and adapt online throughout their lifetime. However, one concern in evolutionary robotics, and thus in online evolution, is scaling to tasks that require higher behavioural complexity [2]. For general-purpose robots capable of learning a variety of different skills for different tasks to become feasible, it is required that they can learn new skills while retaining old skills. However, such an objective remains elusive because robots and artificial agents tend to forget previously learned information in a *catastrophic manner* [34] when multiple skills are required.

One solution to allow robots to *accumulate* skills throughout their lifetime is to rely on modular control systems in which different skills are represented as *building blocks* of the system, and then combined in order to solve more complex tasks. In recent work [35], we have taken the first steps towards this goal by developing an approach in which building blocks are evolved or preprogrammed and then seamlessly specified in the neural structure. The building blocks are optimized together with the neural networks’ weights and topology in a unified manner. Future work should explore the usage of this and similar techniques to enable accumulation of learned behaviours, as well as techniques to effectively evolve complex individual skills [36].

## 5. Conclusion

In this article, we reported successful evolution on neural network-based controllers in real robotic hardware to solve two single-robot tasks and one collective robotics task. Controllers were evolved either from random solutions or from solutions pre-evolved in simulation. In all cases, capable solutions were found in one hour or less. Results showed (i) that the fidelity of the simulation imposes different types of constraints on online evolution in real robots, (ii) that more accurate simulations may lead to higher-performing controllers, and (iii) that completing the optimization process in real robots is an effective approach, even if solutions found in simulation differ from solutions in reality. We furthermore demonstrated the adaptive capabilities of online evolution in real robotic hardware, including a robot collective able to overcome faults injected in the motors of multiple robots simultaneously. To conclude, we showed that each of the main algorithmic components of odNEAT contributes to the algorithm’s performance.

## Supplementary Material

Transferring Simulation-evolved Controllers to Real Robots

## Data accessibility

The raw on-board logs from our experiments and source code are available online on Dryad Digital Repository (http://dx.doi.org/10.5061/dryad.s8t8p) [37].
