# Supplementary material for: Evolutionary online behaviour learning and adaptation in real robots
Source: R Soc Open Sci. 2017 Jul 26;4(7):160938. doi: 10.1098/rsos.160938 (PMC5541525; doi:10.1098/rsos.160938)
Supplement: Transferring Simulation-evolved Controllers to Real Robots [file rsos160938supp1.zip › RSPA_LOGO-color.pdf]

PROCEEDINGS
OF
THE ROYAL SOCIETY
A
