# Supplementary material for: Evolutionary online behaviour learning and adaptation in real robots
Source: R Soc Open Sci. 2017 Jul 26;4(7):160938. doi: 10.1098/rsos.160938 (PMC5541525; doi:10.1098/rsos.160938)
Supplement: Transferring Simulation-evolved Controllers to Real Robots [file rsos160938supp1.zip › supp_author_tex.pdf]

ROYAL SOCIETY
OPEN SCIENCE

rsos.royalsocietypublishing.org

Research

Article submitted to journal

Subject Areas:
robotics, artificial intelligence

Keywords:
online evolution, learning, fault tolerance, real robots

Author for correspondence:
Fernando Silva
e-mail: fsilva@di.fc.ul.pt

Supplementary Material for Evolutionary Online Behaviour Learning and Adaptation in Real Robots

Fernando Silva[1,2,3], Luís Correia[2] and Anders Lyhne Christensen[1,3,4]

[1]Bio-inspired Computation and Intelligent Machines Lab, 1649-026 Lisboa, Portugal
[2]BioISI, Faculdade de Ciências, Universidade de Lisboa, 1749-016 Lisboa, Portugal
[3]Instituto de Telecomunicações, 1049-001 Lisboa, Portugal
[4]Instituto Universitário de Lisboa (ISCTE-IUL), 1649-026 Lisboa, Portugal

© 2017 The Authors. Published by the Royal Society under the terms of the Creative Commons Attribution License http://creativecommons.org/licenses/by/4.0/, which permits unrestricted use, provided the original author and source are credited.

THE ROYAL SOCIETY
PUBLISHING

Text S1 - Transferring Simulation-evolved Controllers to Real Robots

In this section, we assess how the highest-performing controllers found in simulation transfer to real robots. First, we evolved controllers in simulations with varying degree of fidelity by using three complementary methods to model sensory inputs: taking samples from the real robots' sensors [1], introducing a conservative form of noise in simulated sensors [1], and perfect readings, that is, without any stochasticity associated. Using samples from real sensors increases the accuracy of simulations because a more realistic sensor model is employed [1], which in turn has the potential to reduce the difference between the sensory input experienced in simulation and in reality. Conservative noise, on the other hand, has been widely adopted [2] because it has the potential to promote the evolution of robust controllers that can tolerate variations in the sensory inputs profile, including differences between inputs in simulation and in real robots.

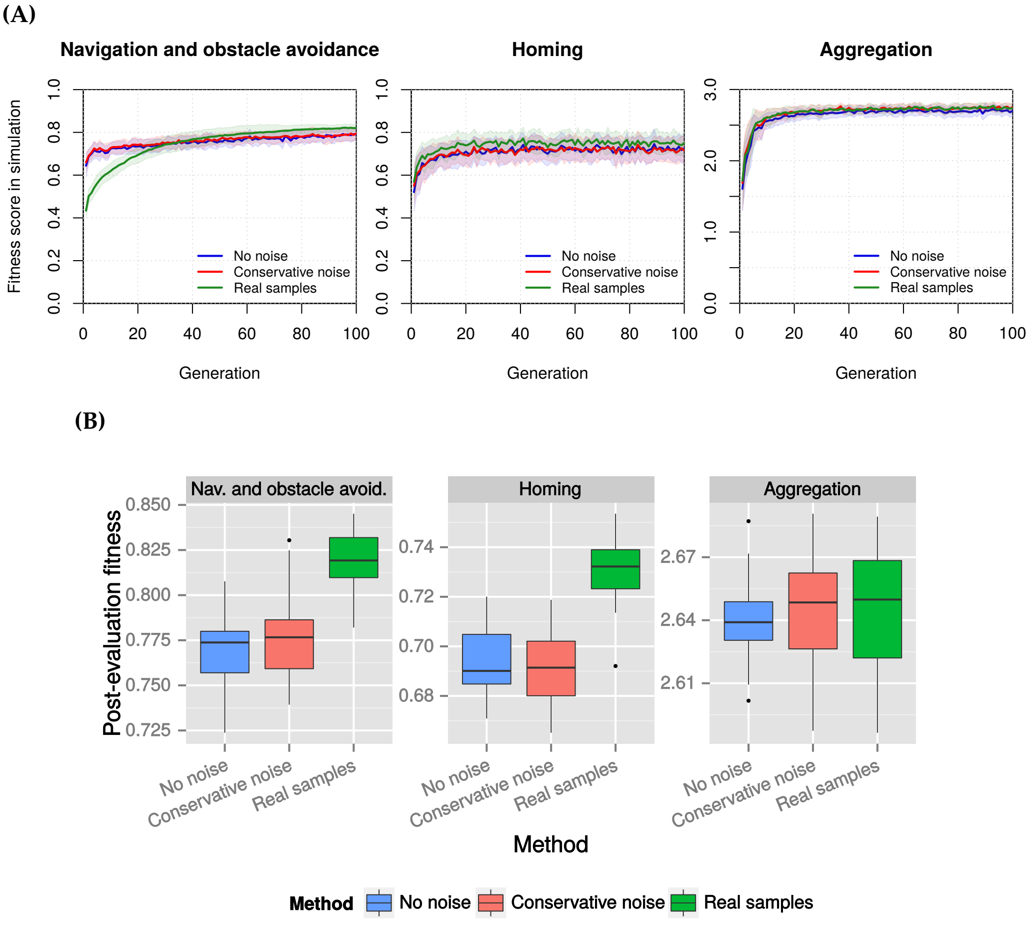

Figure S1: Fitness plots for the simulation-based experiments. **(A)** Highest fitness scores found so far at each generation, for a given sensor modelling approach. The lines depict the mean of 30 runs, with the respective standard deviation shown in lighter colours. **(B)** Distribution of post-evolution evaluation fitness scores for the highest-performing controller of every simulation-based run.

The results of the simulation-based experiments, namely the fitness trajectories and the post-evolution evaluation fitness scores are shown in Fig. S1. Solutions for the three tasks were found within 100 generations for all sensor modelling methods. In the simulation-based post-evolution evaluation, controllers evolved using the real samples method significantly outperform those evolved using the no noise and conservative noise modelling methods in both the navigation and obstacle avoidance, and homing tasks ($\rho < 0.0001$, Mann-Whitney). In the aggregation task, differences in the post-evolution evaluation fitness scores are not significant ($\rho > 0.05$).

Table S1: Controller performance in simulation and in reality. In the navigation and obstacle avoidance task and in the homing task, the fitness scores can take values in the interval [0,1]. In the aggregation task, the fitness scores can take values in the interval [0,3].

Task	Variants	Sim.	Real. (mean)	Real. (median)	Real. ([worst, best])
Navigation	No noise	0.81	0.57	0.58	[0.52, 0.59]
	Conserv. noise	0.83	0.01	0.00	[0.00, 0.04]
	Real samples	0.85	0.81	0.81	[0,79, 0.83]
Homing	No noise	0.72	0.02	0.01	[0.00, 0.04]
	Conserv. noise	0.72	0.02	0.00	[0.00, 0.06]
	Real samples	0.74	0.42	0.49	[0.17, 0.62]
Aggregation	No noise	2.69	0.80	0.60	[0.37,1.44]
	Conserv. noise	2.69	0.81	0.90	[0.36,1.38]
	Real samples	2.69	0.81	0.84	[0.32,1.43]

In real robots, we assessed the highest-performing controller found for each task in simulation, and we compared the performance levels of controllers in simulation and in real robotic hardware. Table S1 shows how well the highest-performing controller found in simulation for a given experimental configuration transfers to real robotic hardware (five real-robot evaluations per controller). In the navigation and obstacle avoidance task, the real samples method is the one that enables the most effective transfer, as controllers yielded similar performance levels in simulation and in reality. Controllers evolved with the conservative noise approach failed to transfer successfully to real robots. Controllers evolved without noise in the simulated sensors transferred from simulation to reality with average fitness decreases of 0.24. In the homing task, the real samples method was also the most effective one, although significant decreases in performance were frequently observed. Controllers evolved with noise and controllers evolved without noise failed when executing on the real robots. Visual inspection of the behaviour revealed that controllers that failed in the navigation task and in the homing task typically could not resume operation after being in close proximity and/or colliding with the walls of the arena. For example, in the homing task, this circumstance caused the failure of evolved behavioural strategies in which the robot moved forward until a wall was in close proximity, and then backwards towards the target area. That is, controllers were not prepared for specific sensory conditions that they encountered in the real environments. Controllers evolved using samples from the real robots are less prone to suffer from this discrepancy. In the aggregation task, the transfer of controllers from simulation to reality consistently resulted in a significant performance degradation.

The results presented above indicate that more accurate simulations, such as those that make use of samples from real robots, typically facilitate better transfer of controllers from simulation to reality. The conservative noise approach, although widely adopted [2], is sensitive to the experimental conditions as it cannot guarantee that the relevant sensorimotor conditions are present in simulation (e.g. specific view of the walls of the arena from the robot's vantage point). Nonetheless, similar issues affect the real samples method as the task becomes more challenging (e.g. when multiple robots are involved). As more types of objects are considered

or richer robot-environment interactions exist in the environments, the more samples have to be taken from a large number of positions because there are multiple unique readings of the objects depending on the robot's relative location and orientation [2]. If such sampling is not performed, it introduces the potential for robots to exploit poorly modelled phenomena, resulting in sub-optimal performance when controllers are deployed to real robots.

Text S2 - Configuration of odNEAT

In the real-robot experiments, odNEAT is configured with the following parameters: internal population - 40, crossover rate - 0.25, mutation rate - 0.4, add neuron rate - 0.03, add connection rate - 0.05, and weight mutation magnitude - 0.5. The control system of each robot executing odNEAT is based on a discrete-time neural network with connection weights in the range [-10,10]. The inputs of the neural network are the readings from the sensors, normalised to the interval [0,1]. The output layer is composed of two neurons. The values of the output neurons are linearly scaled from [0,1] to [-1,1] to set the signed speed of each wheel.

Figure S2 - Behaviour-fitness map for the aggregation task

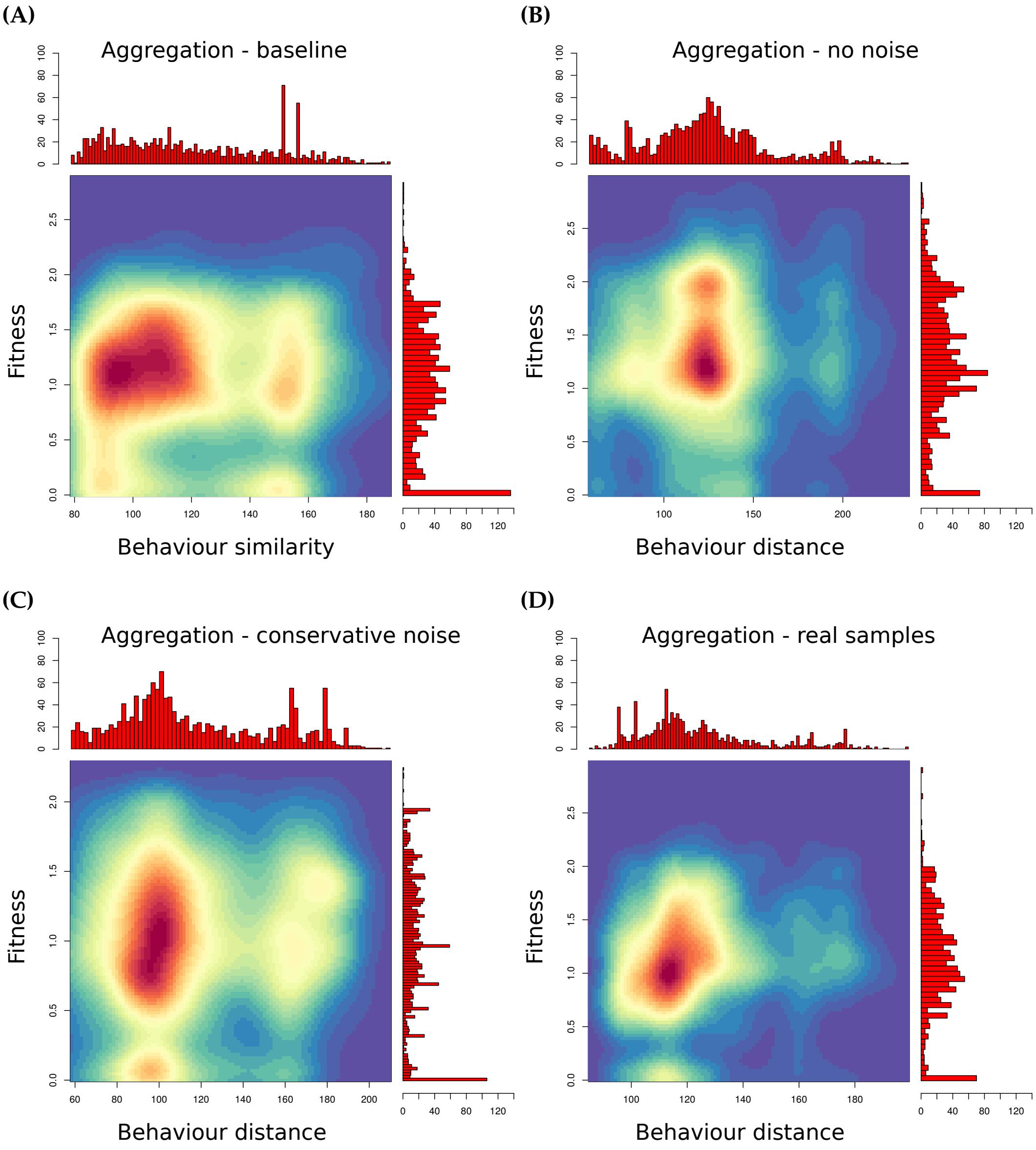

Figure S2: Behaviour-fitness map for the aggregation task. Behaviour distance is relative to the initial controllers: a randomly generated controller for the baseline setup **(A)**, and the seed controller for the no noise **(B)**, conservative noise **(C)**, and real samples setup **(D)**. In general, the lowest-performing method is the conservative noise approach, which is not able to reach the highest-fitness regions (only few solutions with fitness score above 2.0).

Figure S3 - Behaviour-fitness map for the adaptation experiments

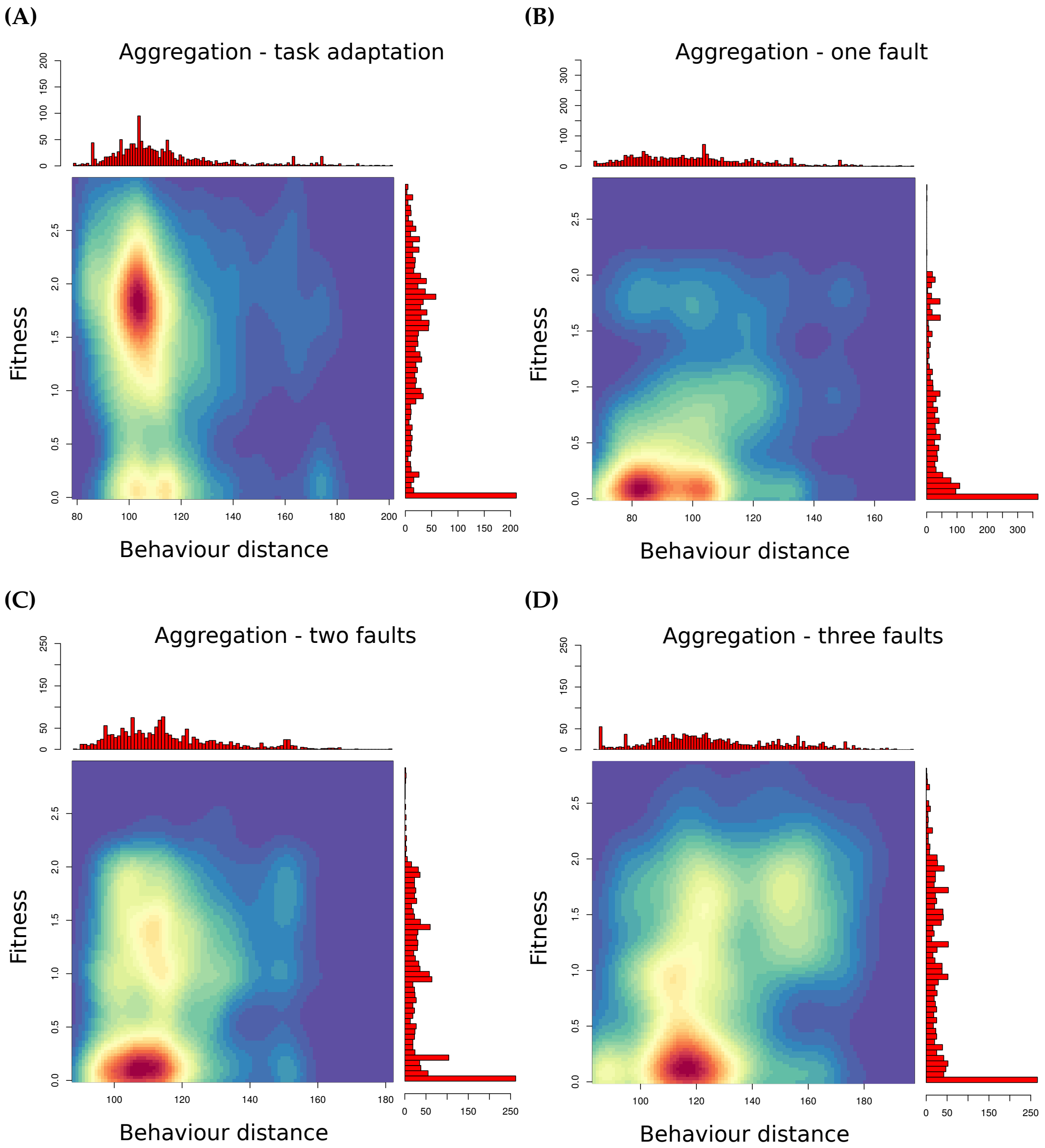

Figure S3: Behaviour-fitness map for the adaptation experiments. Behaviour distance is relative to the controllers that were executing when: the task requirements changed **(A)**, one fault was injected **(B)**, two faults were injected **(C)**, and three faults were injected **(D)**.

References

1. Miglino O, Lund HH, Nolfi S. 1996 Evolving mobile robots in simulated and real environments. *Artif. Life* **2**, 417–434. (doi:10.1162/artl.1995.2.4.417))
2. Silva F, Duarte M, Correia L, Oliveira SM, Christensen AL. 2016. Open issues in evolutionary robotics. *Evol. Comput.* **24**, 205–236. (doi:10.1162/EVCO_a_00172)
