# Supplementary figures and images for: Evolutionary online behaviour learning and adaptation in real robots
Source: R Soc Open Sci. 2017 Jul 26;4(7):160938. doi: 10.1098/rsos.160938 (PMC5541525; doi:10.1098/rsos.160938)

# Aggregation - conservative noise

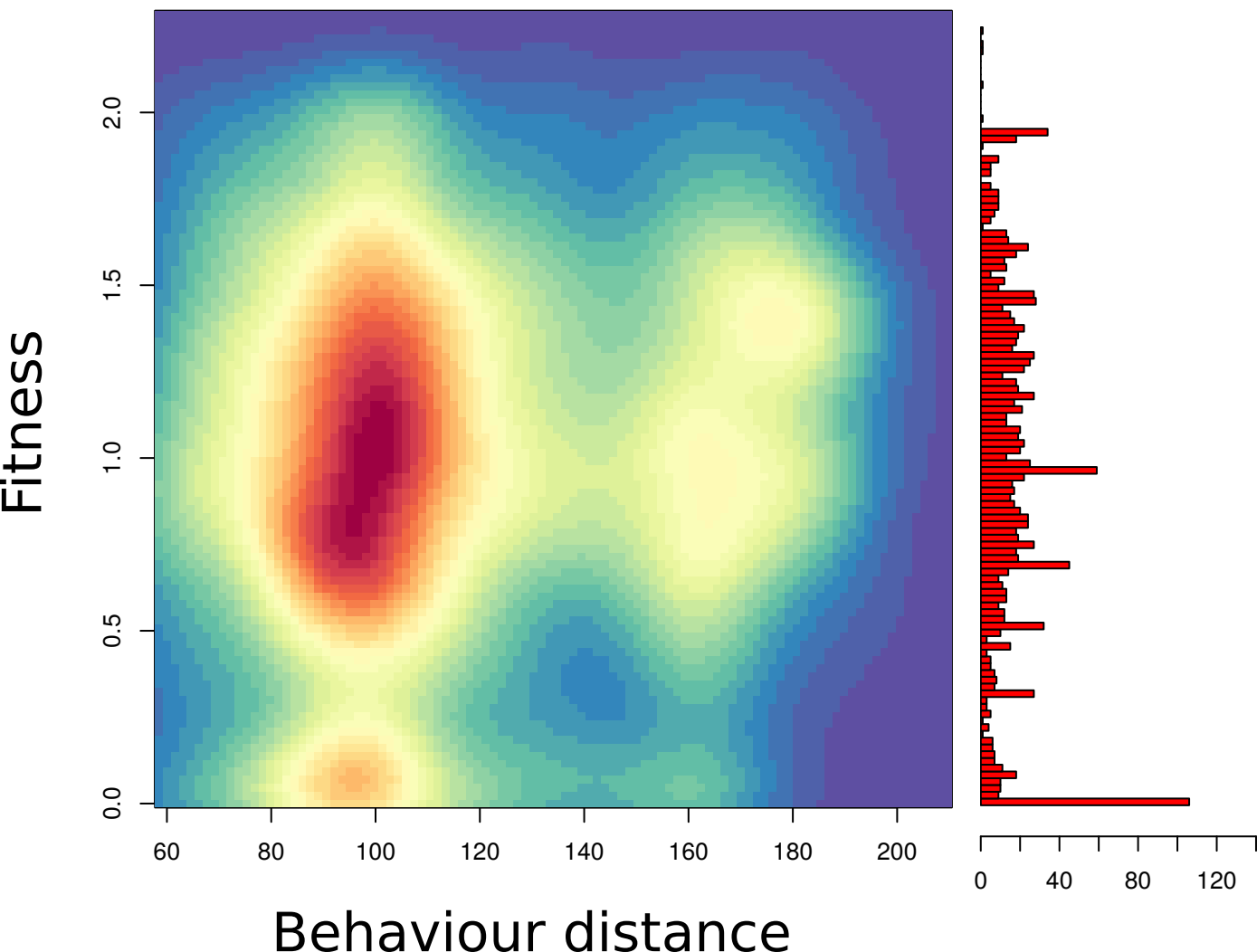

Supplement: Transferring Simulation-evolved Controllers to Real Robots [file rsos160938supp1.zip › figs/combined_bins_aggregation_conservative_noise.pdf]

# Aggregation - real samples

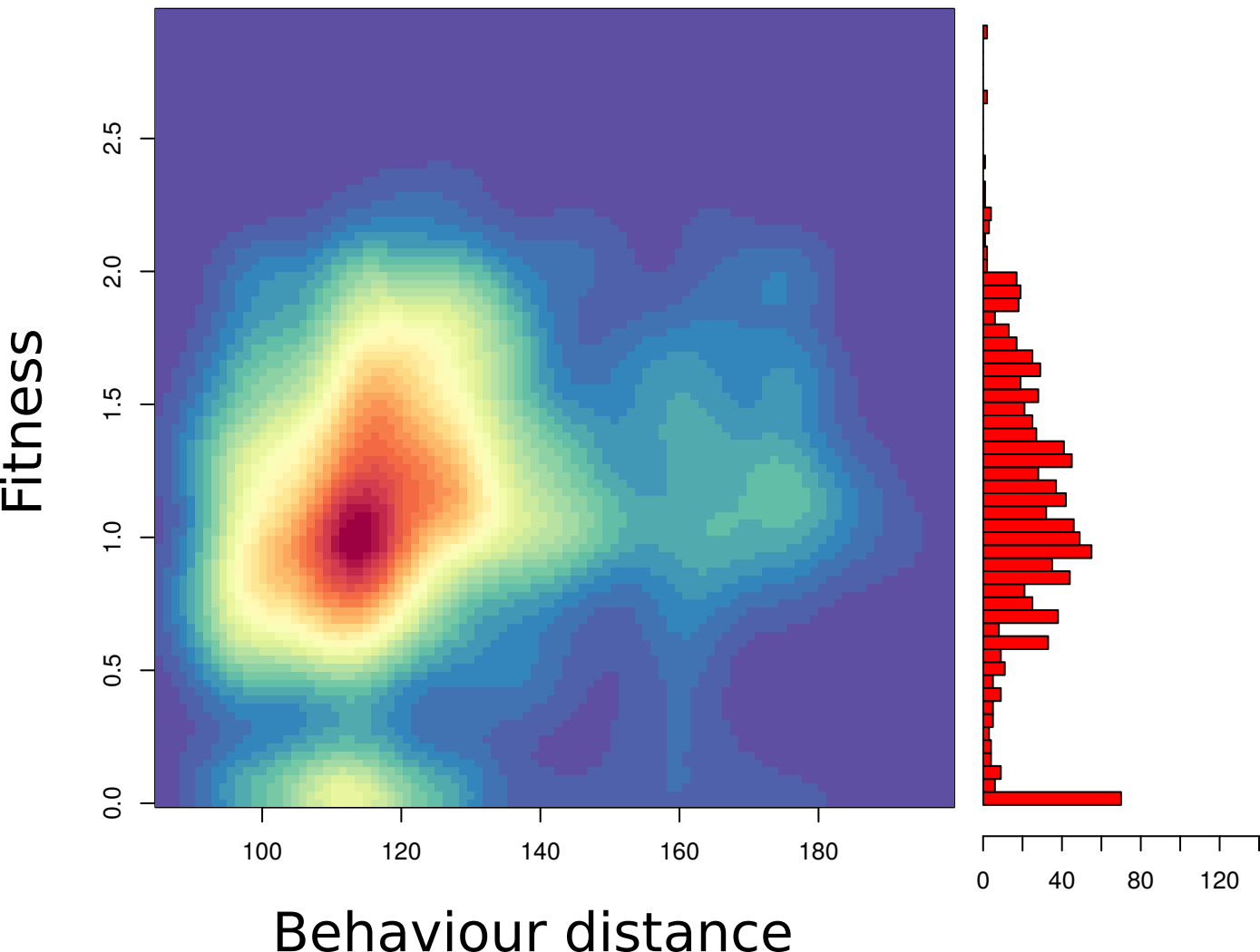

Supplement: Transferring Simulation-evolved Controllers to Real Robots [file rsos160938supp1.zip › figs/combined_bins_aggregation_real_samples.pdf]

# Aggregation - baseline

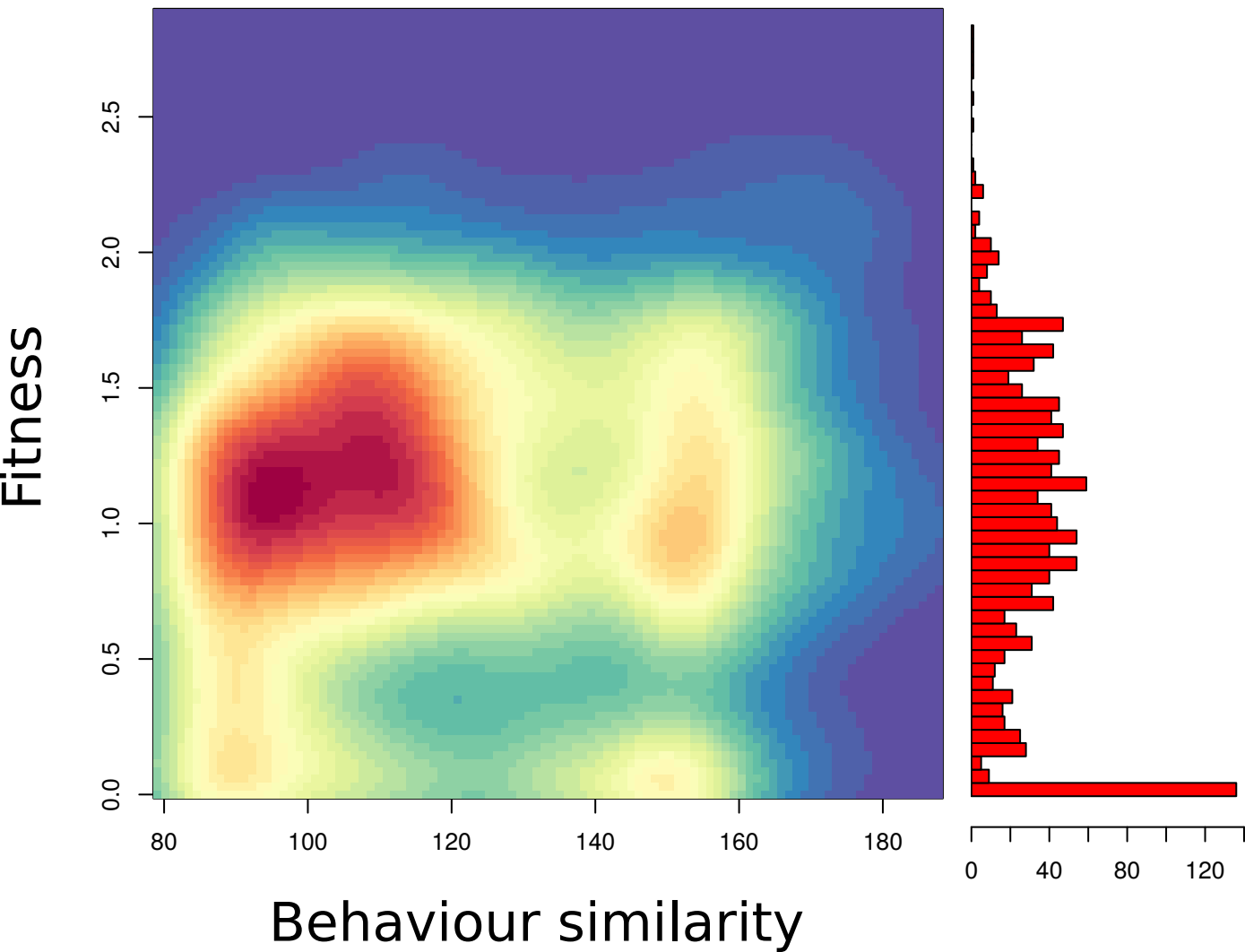

Supplement: Transferring Simulation-evolved Controllers to Real Robots [file rsos160938supp1.zip › figs/combined_bins_aggregation_evo_alone_v2.pdf]

# Aggregation - no noise

Fitness

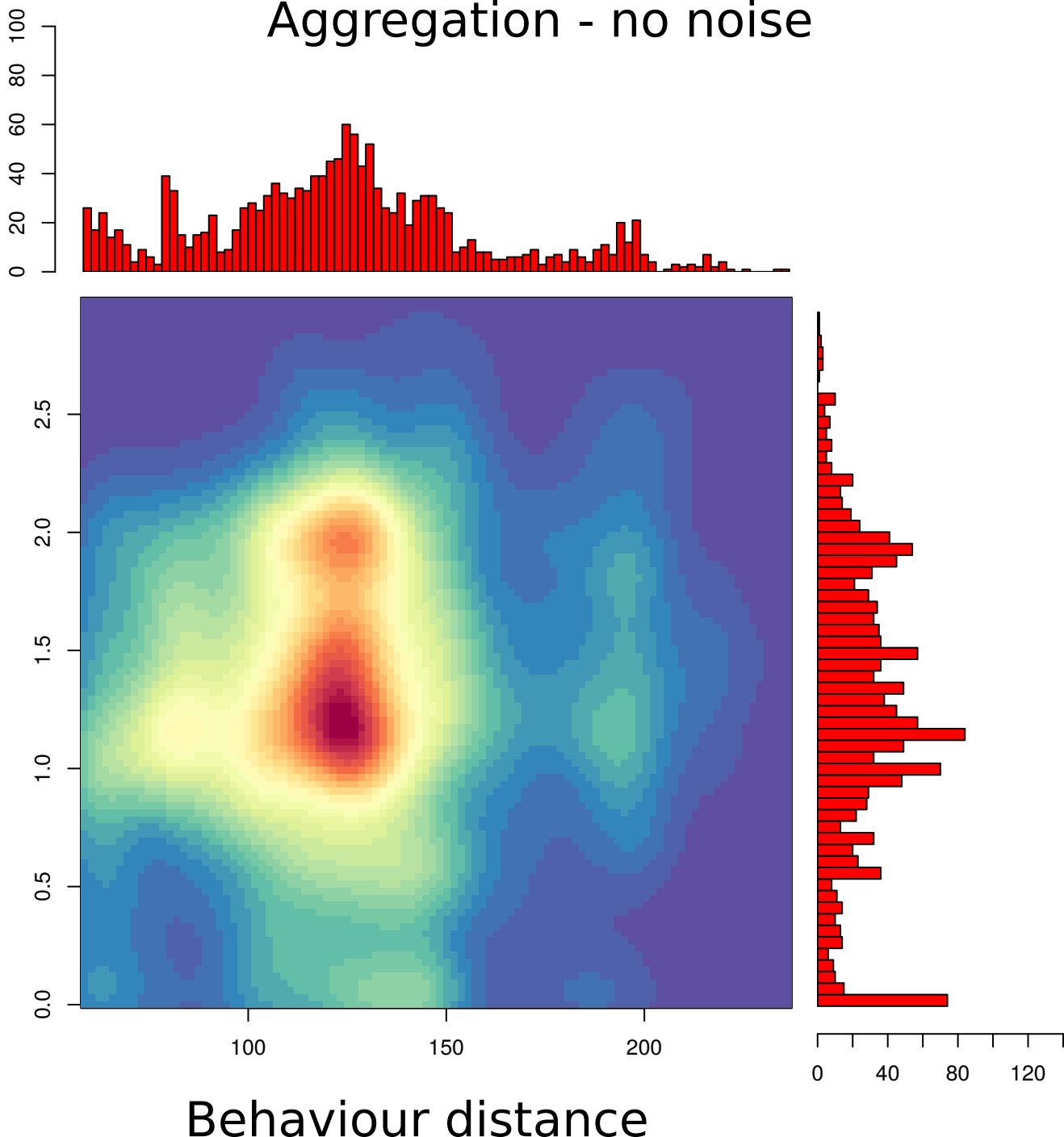

Supplement: Transferring Simulation-evolved Controllers to Real Robots [file rsos160938supp1.zip › figs/combined_bins_aggregation_no_noise.pdf]

# Aggregation - task adaptation

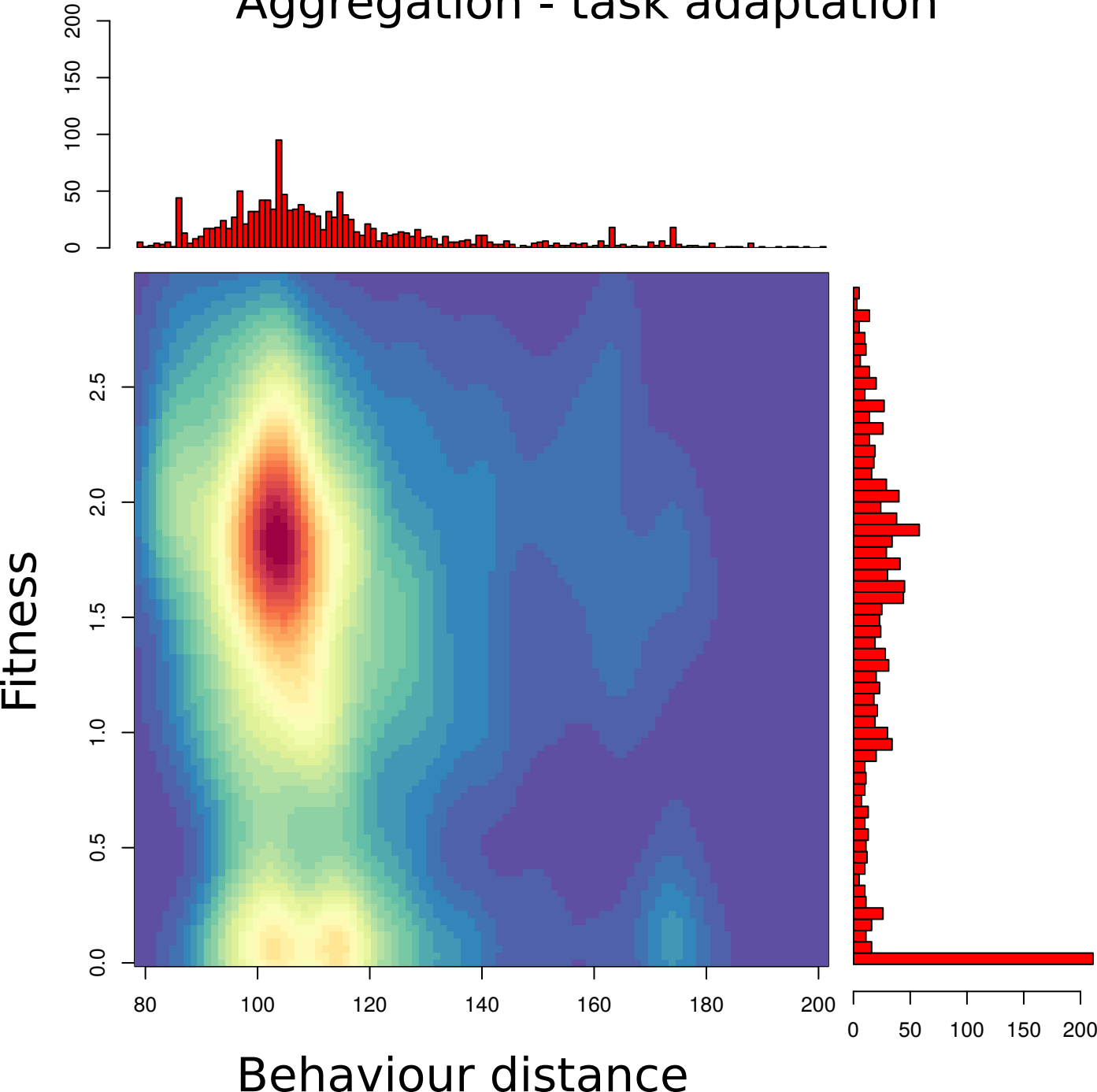

Supplement: Transferring Simulation-evolved Controllers to Real Robots [file rsos160938supp1.zip › figs/combined_bins_task_adaptation.pdf]

### Navigation and obstacle avoidance

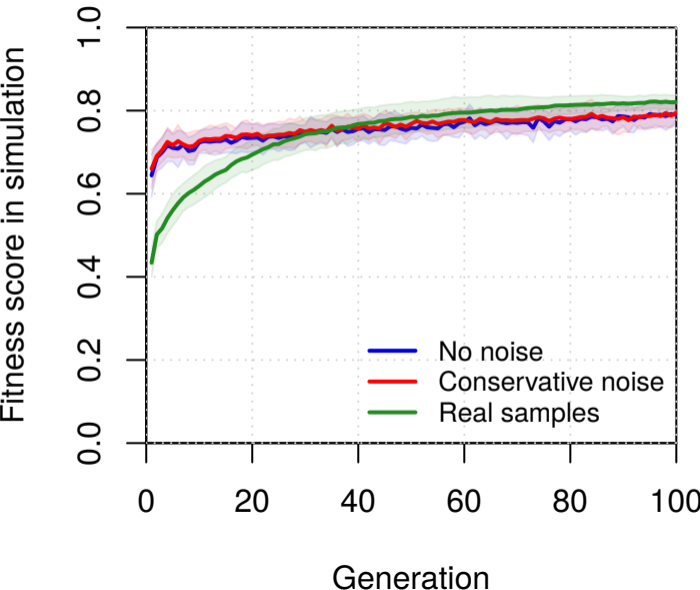

### Homing

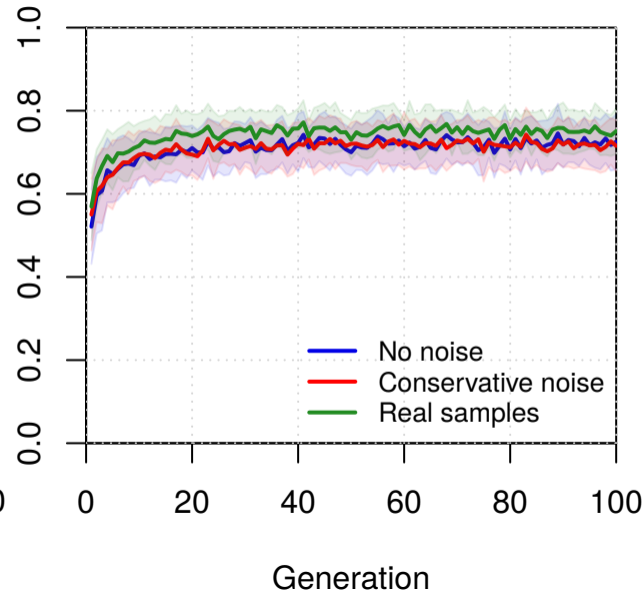

### Aggregation

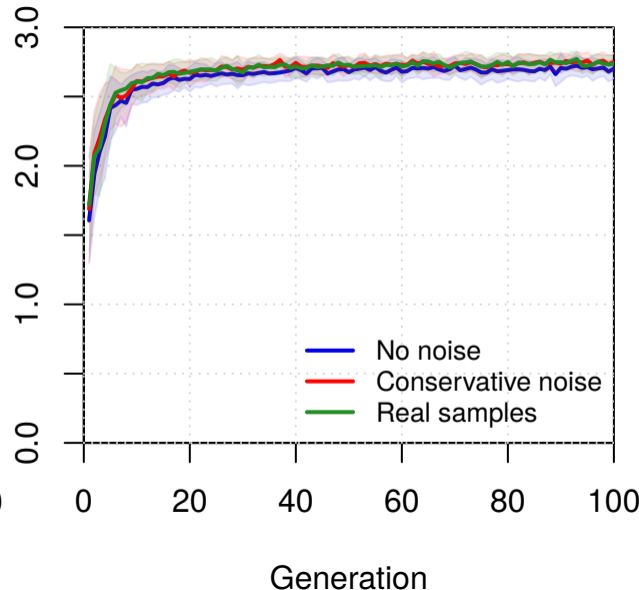

Supplement: Transferring Simulation-evolved Controllers to Real Robots [file rsos160938supp1.zip › figs/combined_simulation_fitness.pdf]

# Aggregation - three faults

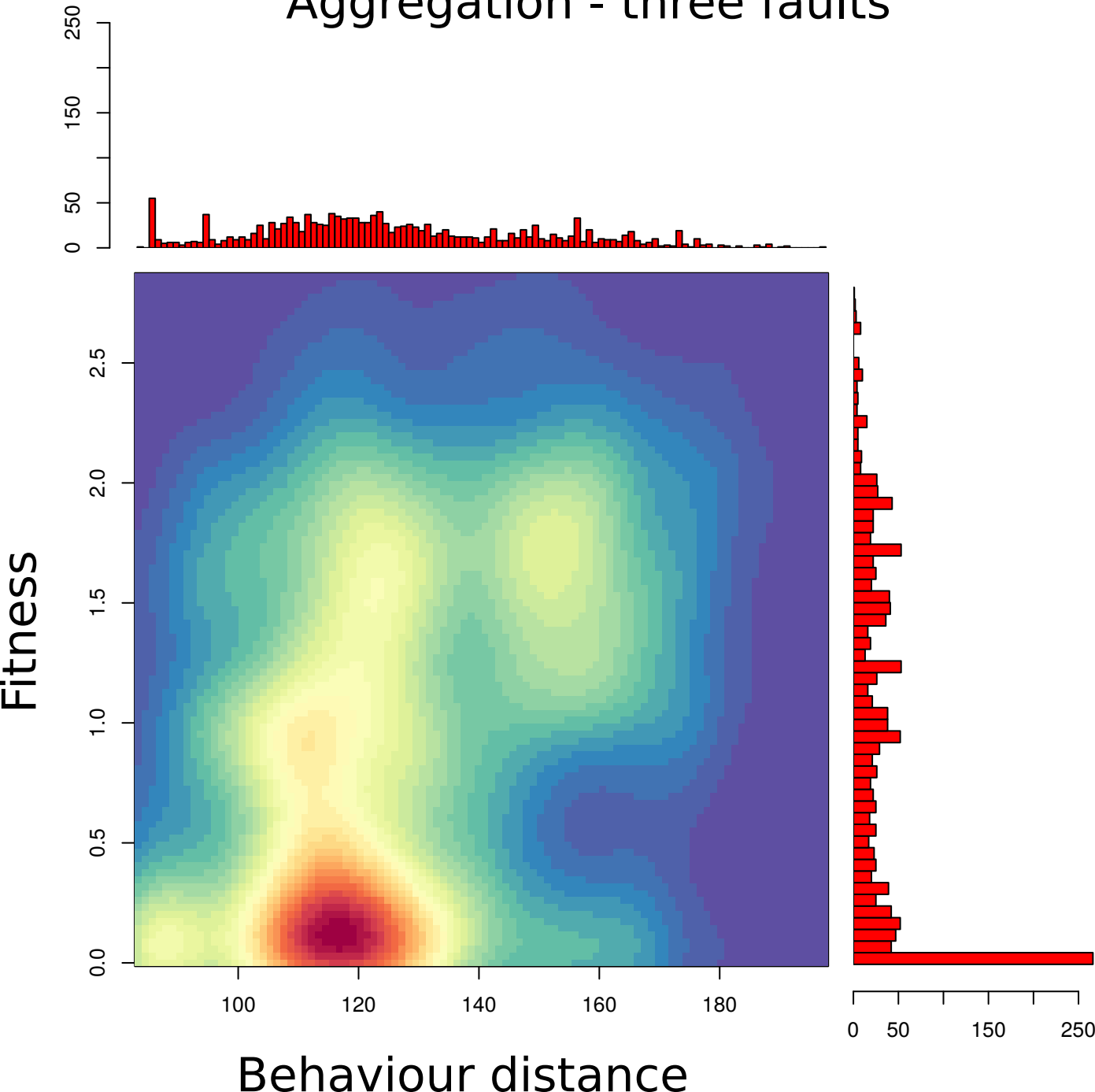

Supplement: Transferring Simulation-evolved Controllers to Real Robots [file rsos160938supp1.zip › figs/combined_bins_three_faults.pdf]

# Aggregation - two faults

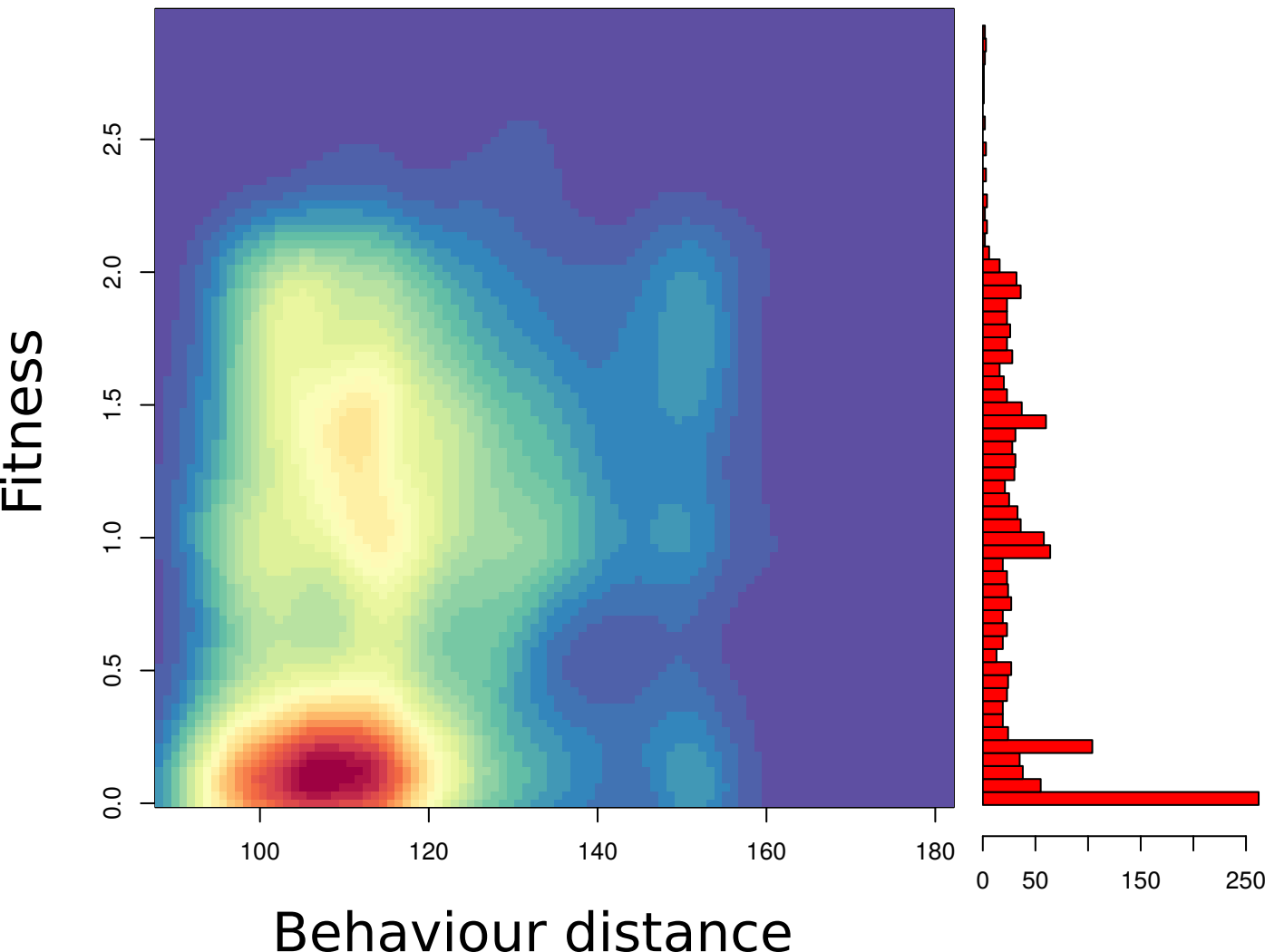

Supplement: Transferring Simulation-evolved Controllers to Real Robots [file rsos160938supp1.zip › figs/combined_bins_two_faults.pdf]

# Aggregation - one fault

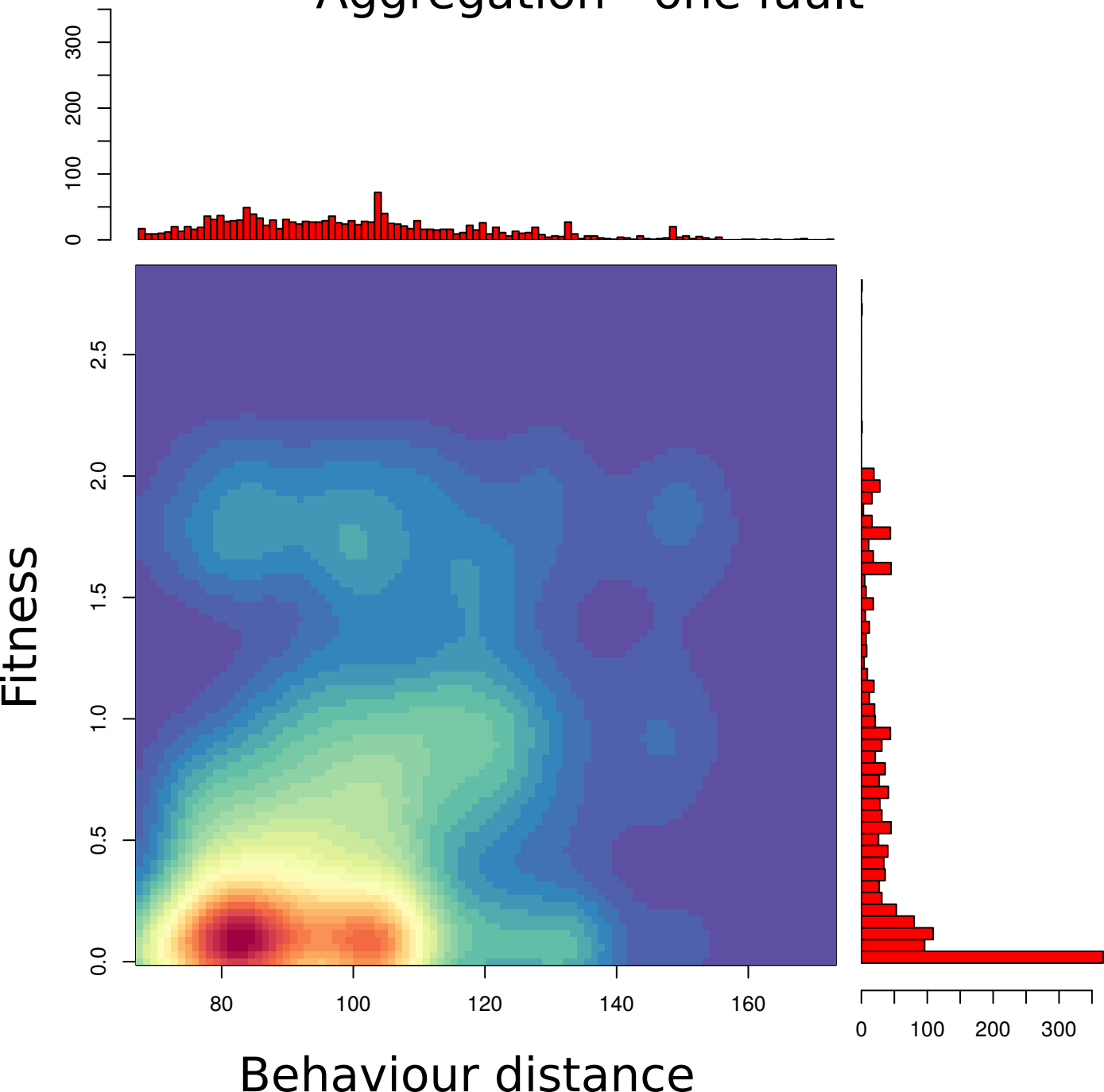

Supplement: Transferring Simulation-evolved Controllers to Real Robots [file rsos160938supp1.zip › figs/combined_bins_one_fault.pdf]

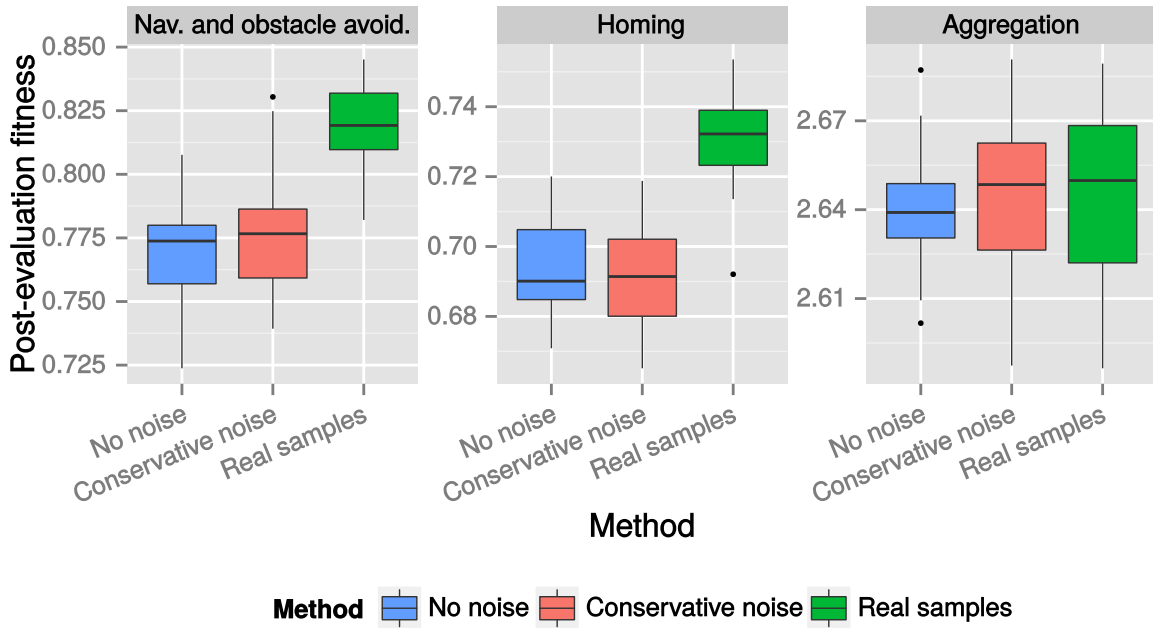

Supplement: Transferring Simulation-evolved Controllers to Real Robots [file rsos160938supp1.zip › figs/plot_bests_simulation2.pdf]

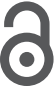

Supplement: Transferring Simulation-evolved Controllers to Real Robots [file rsos160938supp1.zip › openaccesslogo_bw.pdf]

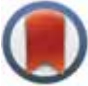

CrossMark

[click for updates](#)

Supplement: Transferring Simulation-evolved Controllers to Real Robots [file rsos160938supp1.zip › RS_crossmark_logo.pdf]

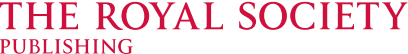

Supplement: Transferring Simulation-evolved Controllers to Real Robots [file rsos160938supp1.zip › RSOS_Pubs_Logo_Line_CMYK.pdf]
